# Exploring health system resilience during the COVID-19 pandemic in Sweden: an interrupted time series analysis of service utilisation and sociodemographic differences

**DOI:** 10.1186/s12913-025-13551-6

**Published:** 2025-10-02

**Authors:** Emily Maresch, My Fridell, Maxim Kan, Zangin Zeebari, Ulf O. Gustafsson, Anna Mia Ekström, Helena Nordenstedt

**Affiliations:** 1 Department of Global Public Health, Karolinska Institute, Stockholm, Sweden; 2 Department of Economics, Finance, Statistics and Informatics, Jönköping University, Jönköping, Sweden; 3 Department of Clinical Sciences at Danderyd Hospital, Karolinska Institutet, Stockholm, Sweden; 4 Department of Molecular Medicine and Surgery, Karolinska Institutet, Stockholm, Sweden; 5 Department of Pelvic Cancer, Division of Coloproctology, Center for Digestive Diseases, Karolinska University Hospital, Stockholm, Sweden; 6 Department of Infectious Diseases/Venhälsan, South General Hospital, Stockholm, Sweden; 7 Department of Medical Specialties, Danderyd Hospital, Stockholm, Sweden

**Keywords:** Health system resilience, COVID-19, Sweden, Pandemic, Essential health services

## Abstract

**Background:**

The COVID-19 pandemic strained health systems worldwide, diverting resources and disrupting routine healthcare services. These disruptions may lead to health risks from delayed or reduced care. Health system resilience (HSR) – a critical factor in maintaining health services during crises – refers to a system’s ability to anticipate, prepare for, absorb, respond to, recover from, and adapt to disruptions. To be effective, HSR must also be equitable, ensuring that all socioeconomic groups have access to healthcare services during crises. Sweden’s approach, which relied on public adherence to government recommendations rather than enforcing restrictions, presents a unique case for studying HSR.

**Aim:**

This study aims to evaluate aspects of HSR in Sweden during the COVID-19 pandemic by analysing changes in essential health service utilisation across different phases of the pandemic, and by examining sociodemographic differences in utilisation by education and sex.

**Method:**

This study utilises interrupted time series analysis to evaluate changes in healthcare service utilisation across various levels of the Swedish health system with stratification by education and sex. Primary care was measured through diabetes diagnoses, emergency care through appendicitis cases, inpatient care through hospital admission for inflammatory bowel disease (IBD), and cancer diagnostics through diagnosis rates. Data were obtained from various population based Swedish health registries through the SWECOV project.

**Results:**

The findings highlight varying degrees of disruptions and resilience across different sectors. Emergency care, primary care, and symptom-based cancer diagnosis showed signs of recovery after an initial drop, whereas cancer screening, was more affected. In the case of inpatient care for IBD, the number of admissions declined, though the length of hospital stays remained unchanged. Education level did not impact healthcare utilisation for most indicators, and differences between men and women were generally small.

**Conclusion:**

The Swedish HSR during the COVID-19 pandemic was challenged but remained intact in most healthcare sectors. The health system on different levels also managed to a large degree cater for the diagnoses covered in this study, largely independent of educational level and sex. As data availability increases with time further research will help gain a deeper understanding of the outcomes of the pandemic on health services unrelated to COVID-19, including the role of education in influencing healthcare utilisation.

## Background

The COVID-19 pandemic disrupted health systems globally, leading to unprecedented challenges in managing both COVID-19 cases and non-COVID-19 health issues. Sweden took a unique approach during the early phase of the pandemic by relying on public adherence to government recommendations, rather than strict lockdowns. While other countries gradually reduced their non-pharmacological interventions (NPIs) later in 2020, Sweden largely maintained its initially moderate strategy throughout the year, making it an interesting case for studying health system resilience [1, 2]. Despite this relatively lenient approach at the start of the outbreak, Sweden recorded the lowest overall excess mortality in the European Economic Area during the pandemic [3, 4].

Sweden had one of Europe’s lowest scores on the Oxford Stringency Index, a composite measure based on indicators such as school closures, workplace closures, and travel bans that provides a systematic way to compare government responses to the pandemic across countries [5]. These relatively less stringent measures may have reduced public fear of the virus itself and of healthcare system strain, encouraging people to seek care for non-COVID-19 conditions. However, this approach may in turn also have led to higher COVID-19 transmission rates and increased strain on the healthcare system due to greater mobility.

The World Health Organisation (WHO) defines a health system as all organisations, people, and actions aimed at promoting, restoring, or maintaining health [6]. Health system resilience refers to the ability to withstand and recover from crises, including anticipating risks, preparing for disruptions, and adapting to lessons learned [7, 8]. The COVID-19 pandemic underscored the importance of resilience, especially as it led to increased patient volumes and disrupted routine care [9] potentially out-crowding regular health care that in many instances was temporarily interrupted or postponed. Maintaining essential services, such as elective procedures and screenings, is vital for mitigating long-term health consequences [7].

Sociodemographic factors, such as education, significantly influence access to healthcare services, and the pandemic has exacerbated existing disparities in healthcare utilisation across different socioeconomic status (SES) groups globally [10, 11]. Individuals in lower education groups may have encountered greater barriers to healthcare access, such as limited information on protective measures or, for example, reliance on public transportation, which increased the risk of exposure to the virus [12, 13]. Consequently, the impact of the pandemic on healthcare systems may have varied depending on SES, adding another layer of complexity to understanding the pandemic’s overall effect on healthcare access and utilisation. Sweden experiences inequalities in life expectancy, and mental healthcare utilisation based on educational level and these inequalities seem to be on the rise [14, 15]. However, other research suggests that these disparities are not as prominent in other types of healthcare services such as elderly care [16].

Ensuring equitable resilience is crucial, as socioeconomic disparities and sex can exacerbate the impact of disruptions on vulnerable groups [17]. Research on the pandemic’s impact in high-income countries like Sweden is limited [18, 19], with some studies originating from neighbouring Denmark [20, 21]. However, Denmark implemented a markedly different pandemic strategy compared with Sweden, relying more on strict restrictions rather than voluntary public adherence. Most other studies have focused on low- and middle-income countries, where severe disruptions were reported [22–24].

To our knowledge, no studies provide a comprehensive overview of health system resilience in Sweden, and the effect of education and sex, during the COVID-19 pandemic, particularly given Sweden’s unique approach of relying on public adherence to government recommendations rather than strict restrictions.

## Methods

### Aim

This study aims to fill this gap by evaluating the utilisation of health services across four sectors, and how it might have differed depending on educational level and sex: (i) primary care, (ii) inpatient care, (iii) emergency care, and (iv) cancer diagnostics – offering insights into how Sweden’s health system can inform global strategies for maintaining essential healthcare services during crises.

### Study design

The primary method used for this analysis is interrupted time series (ITS) analysis, a quasi-experimental study design. Data were collected from SWECOV, a comprehensive infrastructure linking multiple Swedish registers and capturing nearly the entire population, before, during and after the pandemic as defined below.

### Data sources

Data was obtained through the SWECOV project, which combines 53 population registers with individual-level data covering 15.7 million Swedish residents (alive 1990–2022) for the purposes of COVID-19 related research [25]. Different registers are linked using a pseudo anonymised key derived from the Swedish personal number, which is generated by Statistics Sweden [26]. Data analysis was conducted via the Microdata Online Access (MONA) portal [27].

The time periods for our analysis were defined as follows: (i) the pre-pandemic period: January 1st, 2018, to March 10th, 2020, (ii) the pandemic period: March 11th, 2020, to February 8th, 2022, and (iii) the post-pandemic period: February 9th, 2022, to December 31st, 2022. The pandemic period was defined based on the declaration of the COVID-19 pandemic in March 2020 by the WHO [28] and the end of the pandemic period was marked by the removal of most recommendations and restrictions due to the pandemic in Sweden on February 9th, 2022 [29]. The new approach, after the 9th of February 2022, shifted to specifically focusing on protecting individuals in healthcare and elderly care with a higher risk of severe illness from COVID-19. As a result of this shift, the statistics on confirmed cases after this date are not comparable to earlier data, marking a significant transition in how the pandemic was monitored and managed. We acknowledge that excess mortality persisted into the period we define as post-pandemic, and that the pandemic phase itself could be further divided into pre- and post-vaccine availability. For this analysis, however, we chose to define the periods based on government regulations and restrictions rather than the timing of pharmaceutical interventions.

### Indicators and variables

The study analyses the utilisation of services of different health system sectors: primary care, inpatient care, emergency care, and cancer diagnostics. For each sector variables were predefined and selected based on previous literature as well as on data availability to represent that specific sector, and no clear link to COVID-19 infection.

For Primary care, we assessed number of new type 2 diabetes diagnoses from 2018 to 2020, using the first prescription of oral antidiabetic drugs (excluding insulin, but including GLP-1 receptor agonists) from 2017 onward, identified through the ATC code A10B, based on data from the Prescribed Drug Register (PDR) [30]. Individuals who had already received a prescription for oral antidiabetic drugs in 2017 were excluded from this analysis, ensuring that only those with a new diagnosis in the subsequent years (2018–2020) were included. This indicator has been used in previous studies as a proxy for primary care utilisation during the pandemic in other settings [22–24]. Due to changes in the guidelines in autumn of 2020, where one peroral antidiabetic (dapagliflozin) was approved for use for “symptomatic heart failure with reduced ejection fraction, even without diabetes” data past October 2020 was excluded from the analysis [31].

For Inpatient Care, we analysed both the average length and number of hospitalisations for IBD patients from 2018 to 2022, using data from the National Patient Register (NPR) [32]. IBD admissions have to our knowledge, not been used previously as an indicator for health system resilience but was chosen in this study to represent a diagnosis that should not be directly affected by COVID-19 infections.

To represent Emergency Care, number of appendicitis diagnoses from 2018 to 2022, extracted from the NPR was chosen. Appendicitis is a condition that typically requires emergency care. Appendicitis as an indicator has been used in a previous study to assess the capacity of emergency care during the pandemic in South Korea [33].

Finally, to study how the health system managed to maintain Cancer Diagnostics, the number of new diagnoses of two different types of cancer was assessed, cervical and oesophageal cancer. These diagnoses, spanning from 2018 to 2022, are based on data from the National Cancer Register (NCR) to which reporting is mandatory [34, 35]. These two types of cancer were chosen because their diagnostic processes represent two different sectors of the health system: screening-based for cervical cancer and symptom-based for oesophageal cancer. In Sweden cervical cancer screening is offered to women aged 23–70 [36]. Cervical cancer has been used in previous studies to assess cancer diagnostics’ health system resilience [22, 23, 37]. For oesophageal cancer, multiple parts of the health system need to be functioning for a diagnosis to be made; primary or emergency care where a patient presents with symptoms, endoscopy services to investigate those symptoms, and pathology services to make a final histological diagnosis of a biopsy taken during the endoscopy.

SES was measured through a proxy: education level. The longitudinal integrated database for health insurance and labour market studies (LISA) provided data on education level from one to seven, where one is “Less than 9 years of education” and 7 is “doctoral education”. For this analysis we chose to use 2 categories: low and high education. Low education comprised Less than 9 years of education (e.g., incomplete compulsory school) or 9–10 years of education (e.g., completed compulsory school) or upper secondary education (2–3 years, e.g., gymnasieskola) or post-secondary education of less than 2 years. Whereas high education included: Post-secondary education of 2 or more years without a degree, post-secondary education with a degree (e.g., bachelor’s or master’s), or doctoral education (PhD or equivalent) [38, 39]. Sex was extracted from the Total Population Register.

### Data adjustment and visualization

Data for diabetes first prescriptions and cervical cancer diagnoses were manually adjusted for seasonality, as both conditions exhibit annual fluctuations in diagnosis rates, likely due to changes in healthcare utilisation and diagnostic practices during holiday periods. Moving averages were applied to some indicators in the graphical representations to effectively illustrate the results.

### Data analysis

Graphical representations of the data were created to facilitate interpretation of changes over time, with tables summarising average utilisation and percentage changes before, during, and after the pandemic, stratified by education and sex. Education was not included for appendicitis, as a large proportion of patients were minors who had not yet completed their education. Structural breaks between the three different time periods, pre-pandemic, pandemic, and post-pandemic were tested using the Wald test to determine significant shifts. The Wald test was used to evaluate the null hypothesis of no structural break to determine whether any changes at the beginning or at the end of the pandemic are statistically significant compared to normal fluctuations throughout the years.

## Results

### Primary care (new prescriptions for diabetes type 2)

**Fig. 1:**
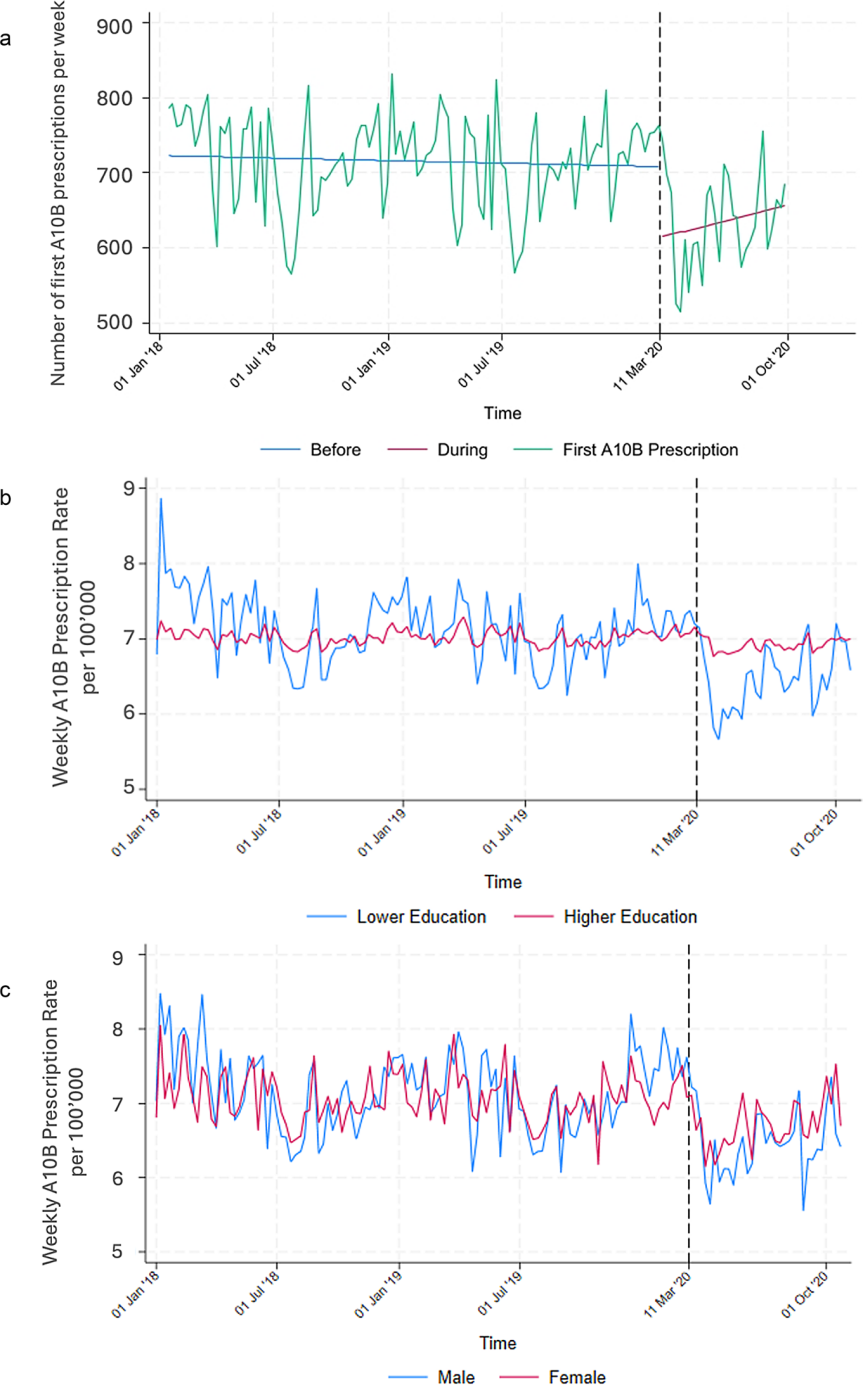
**a** First A10B prescriptions adjusted for seasonality (January 2018 to October 2020, window size 30). The “A10B Prescriptions” line represents seasonally adjusted weekly A10B first prescriptions, while the other two lines represent regressions of the periods before and during the pandemic. The dotted line represents the beginning of the pandemic. **b** First A10B prescriptions adjusted for seasonality (January 2018 to October 2020, window size 30) by education group. **c** First A10B prescriptions adjusted for seasonality (January 2018 to October 2020, window size 30) by sex. The “A10B Prescriptions” line represents seasonally adjusted weekly A10B first prescriptions

At the start of the pandemic, prescriptions for oral antidiabetic medications and GLP-1 analogues dropped substantially, indicating a decline in primary care utilisation. However, by autumn 2020, the number of prescriptions had nearly returned to pre-pandemic levels. (Fig. 1; Table 1)

When dividing the population by education group the results show some differences. People of lower education level utilised the primary care system less for first A10B prescriptions as the pandemic began. People in this group are also more sensitive to seasonal trends, especially during the summer (Fig. 1b).

There are no strong differences between men and women when it comes to A10B first prescription. Prescriptions went down for both groups, slightly more for men than for women (Fig. 1c).

### Inpatient care (IBD admissions)

**Fig. 2:**
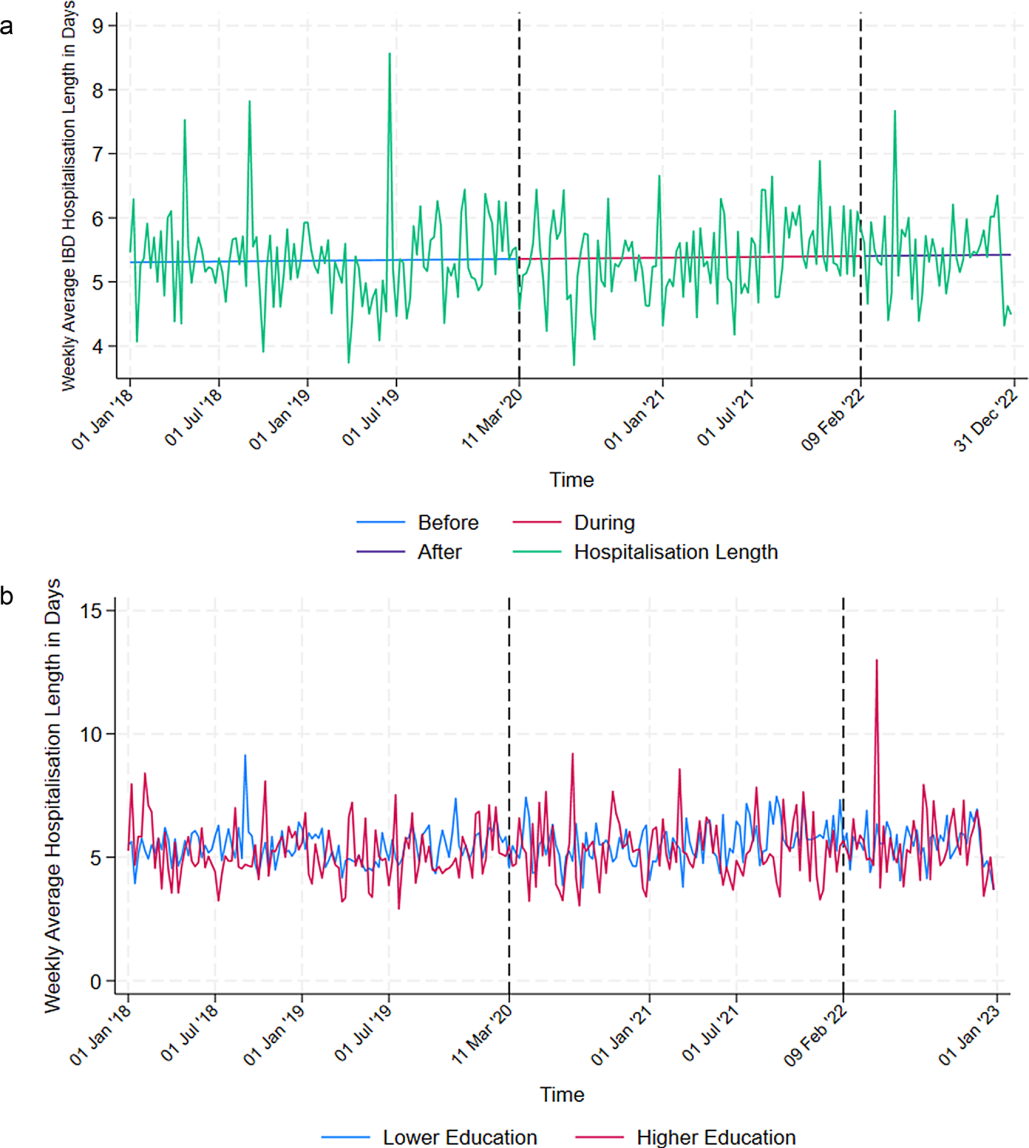
**a** Weekly average IBD hospitalisation length in days (2018-2022, window size 30). The dotted lines mark the beginning and the end of the pandemic. **b** Weekly average IBD hospitalisation length in days by education group (2018–2022, window size 30). The dotted lines mark the beginning and the end of the pandemic

The number of days spent in any hospital for IBD showed no significant changes over time before, during, or after the pandemic (Fig. 2a; Table 1.)

The length of hospitalisation for IBD was similar, independent on level of education (Fig. 2b).

IBD hospitalisation length is no different in men and women throughout the entire observation period and showed no changes over time (data not shown).

**Fig. 3:**
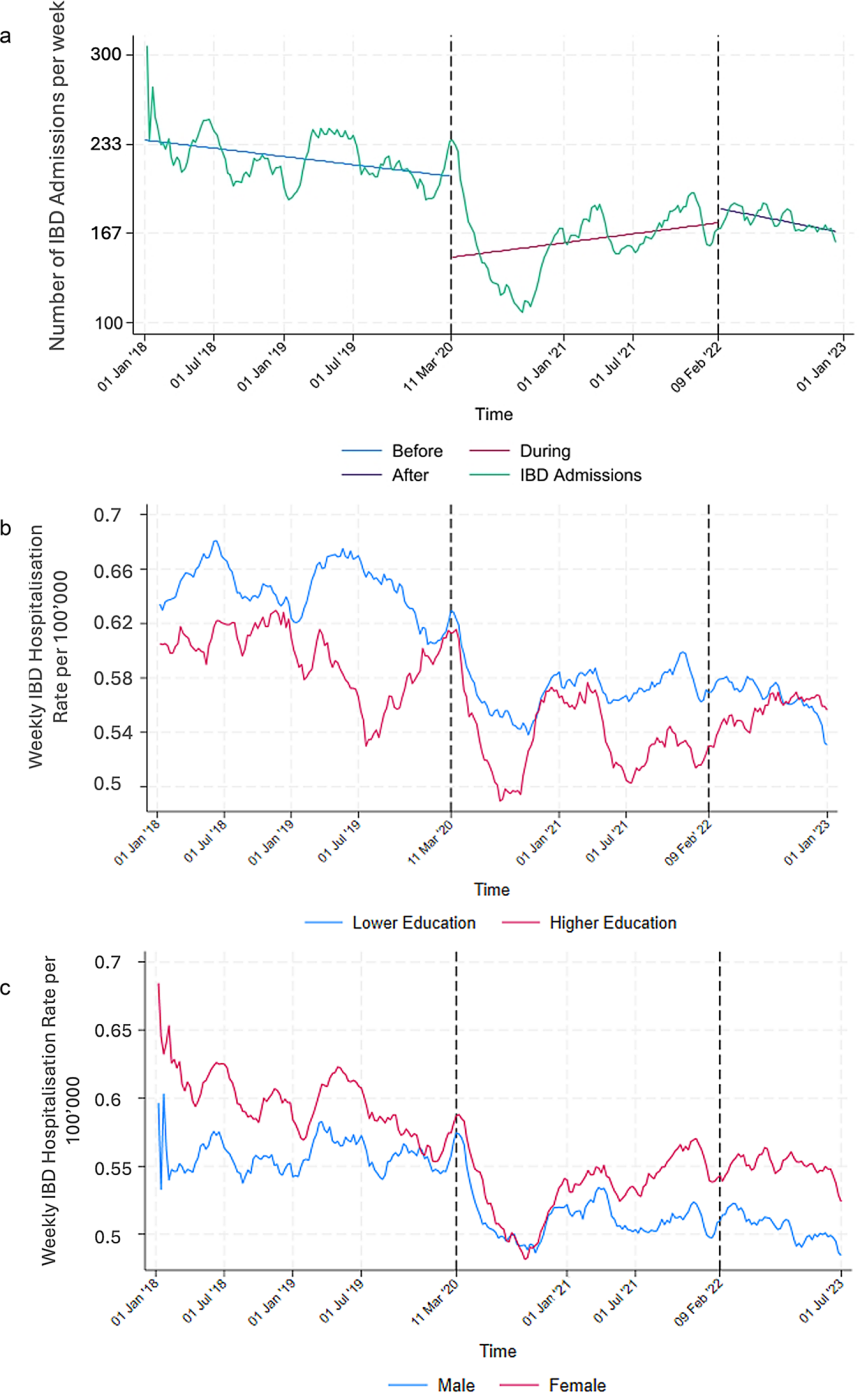
**a** Moving average of number of weekly IBD related admissions (2018–2022, window size 30). The dotted lines mark the beginning and end of the COVID-19 pandemic. **b** Moving average of the rate of weekly IBD related admissions by education group (2018–2022, window size 30). The dotted lines mark the beginning and end of the COVID-19 pandemic. **c** Moving average of the rate of weekly IBD related admissions by sex (2018–2022, window size 30). The dotted lines mark the beginning and end of the COVID-19 pandemic

However, the number of weekly hospital admissions of IBD patients decreased significantly at the start of the pandemic and did not fully recover in the post-pandemic period, possibly continuing a decreasing trend seen already before the pandemic. (Fig. 3, and Table 1).

The overall rate of the higher education group is lower when it comes to IBD admissions. Both lower and higher education group experienced a drop at the start of the pandemic, although it was more pronounced in the higher education group (Fig. 3b).

IBD admissions are over time higher in women than in men, excluding a small dip in the end of 2020 when women’s admissions dip lower than those of men. Neither group has recovered and returned to pre-pandemic levels by the end of the observation period (Fig. 3c).

### Emergency care (appendicitis diagnoses)

**Fig. 4:**
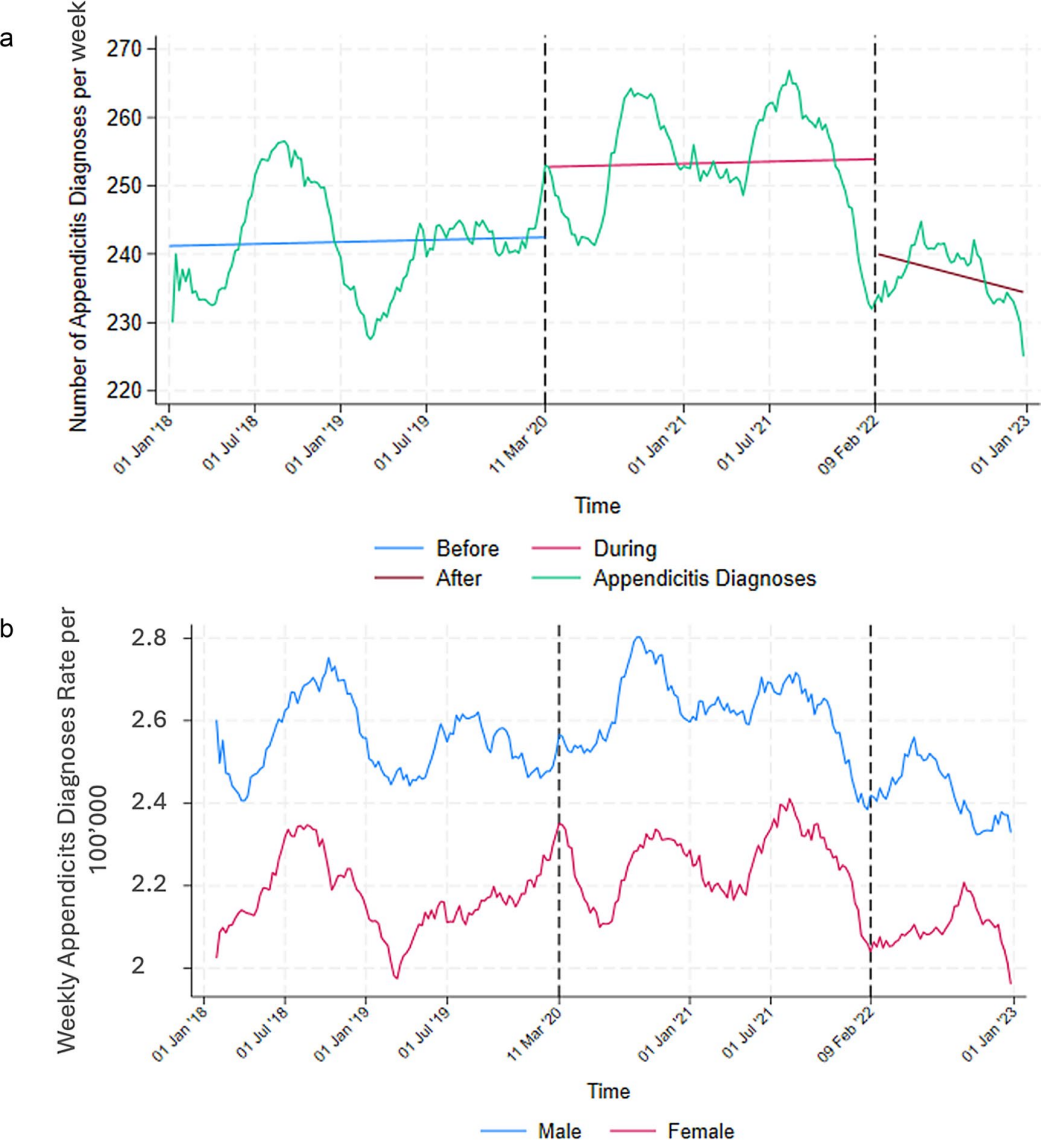
**a** Moving average of appendicitis diagnoses over time (2018–2022, window size 30). The dotted lines mark the beginning and the end of the COVID-19 pandemic. **b** Moving average of appendicitis diagnoses rate over time by sex (2018–2022, window size 30). The dotted lines mark the beginning and the end of the COVID-19 pandemic

During the pandemic, there was an increase in the number of appendicitis diagnoses followed by a substantial decline by the end of the pandemic, with numbers returning to pre-pandemic levels. The number of appendicitis diagnoses per day ranges from a low of 32 (pre- and post-pandemic) to a peak of 38 diagnoses per day (pandemic period). (Fig. 4; Table 1).

Appendicitis cases are throughout the study period higher in men than in women but pattern over time is similar for both sexes. (Fig. 4b).

### Cancer diagnostics (cervical and oesophageal cancer diagnoses)

**Fig. 5:**
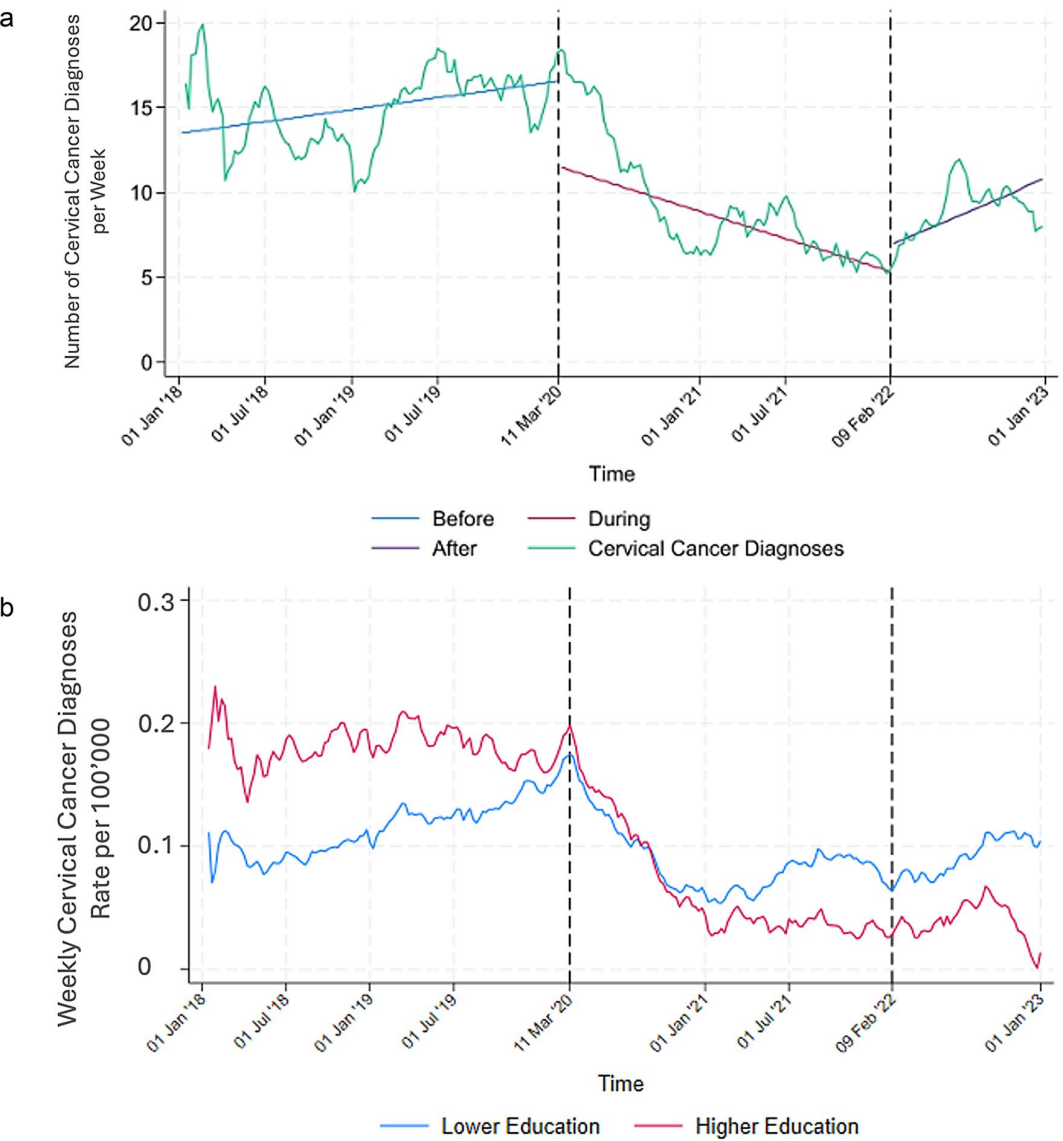
**a** Seasonally adjusted moving average of weekly cervical cancer diagnoses (2018–2022, window size 30). The dotted line marks the beginning of the COVID-19 pandemic. **b** Seasonally adjusted moving average of weekly cervical cancer diagnoses rate by education group (2018–2022, window size 30). The dotted line marks the beginning of the COVID-19 pandemic

Number of diagnoses of cervical cancer per week declined during the pandemic. While there were signs of recovery after February 2022, the observed increase was insufficient to account for all the missed diagnoses during the pandemic (Fig. 5; Table 1).

There was a decrease in number of cervical cancer diagnoses in both the higher and lower education groups. Towards the end of 2020 the diagnosis rate is still lower than before the pandemic in both groups, but the lower education group demonstrates a higher rate of diagnoses than the higher education. Neither group recovers by the end of the observation period (Fig. 5b).

**Fig. 6:**
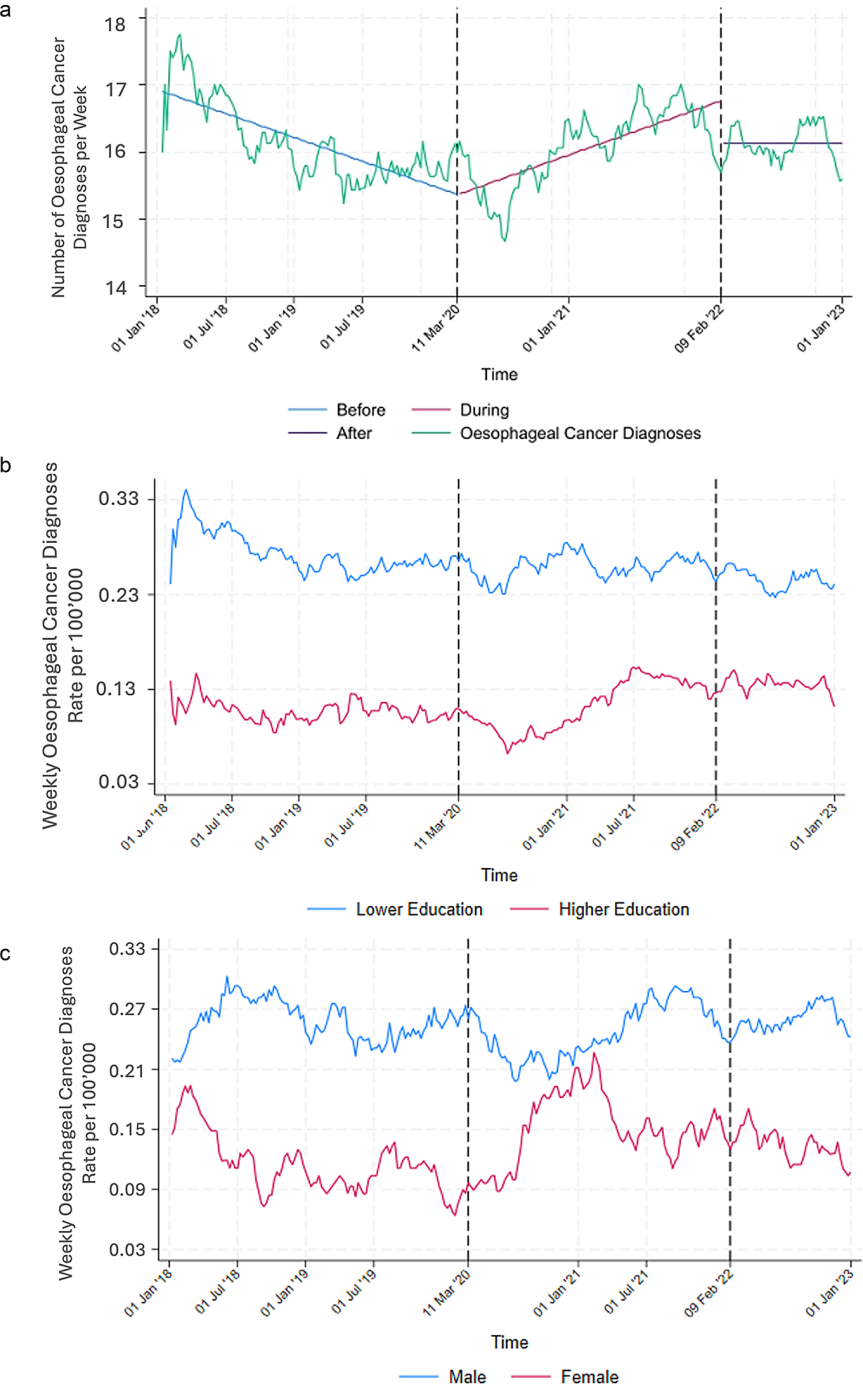
**a** Moving average of weekly oesophageal cancer diagnoses (2018–2022, window size 30). The dotted lines mark the beginning and the end of the pandemic. **b** Moving average of weekly oesophageal cancer diagnoses by education group (2018–2022, window size 30). The dotted lines mark the beginning and the end of the pandemic. **c** Moving average of weekly oesophageal cancer diagnoses by sex (2018–2022, window size 30). The dotted lines mark the beginning and the end of the pandemic

Following a decline in oesophageal cancer diagnoses shortly after the pandemic began, recovery started within a few months. The number of diagnoses then remained relatively stable throughout the post-pandemic period. (Fig. 6; Table 1).

For oesophageal cancer there is a decrease in diagnoses in both higher and lower education group. The higher education group has an overall lower incidence of cases (Fig. 6b).

The incidence of oesophageal cancer is higher in men than in women. At the beginning of the pandemic there is a small decrease in cases diagnosed in men that then recovers by mid 2021. In women there is an increase in diagnoses between mid 2020 and the first quarter of 2021 (Fig. 6c).

### Wald test

The Wald test fails to reject the null hypothesis for IBD hospitalisation length, showing no significant change between the pre-pandemic and pandemic periods, or between the pandemic and post-pandemic periods. It also fails to reject the null hypothesis of no structural break at the end of the pandemic for the number of IBD admissions, and cervical and oesophageal cancer diagnoses. However, the Wald test rejects the null hypothesis for all the other indicators meaning that the structural breaks are statistically significant (Table 1.)

**Table 1:** Wald test testing for structural break at start (11th of March 2020) and end (9th of February 2022) of the pandemic

	Pre-pandemic vs. pandemic		Pandemic vs. post-pandemic	
	Chi2	Wald test	Chi2	Wald test
Diabetes type 2, new diagnoses	6.035	*P* < 0.04*		
IBD length of admissions	0.518	*P* < 0.772	2.018	*P* < 0.365
IBD number of admissions	14.643	*P* < 0.0007*	2.017	*P* < 0.159
Appendicitis diagnoses	26.4	*P* < 0.0000*	29.7330	*P* < 0.0000*
Cervical cancer diagnoses	8.058	*P* < 0.018*	1.69	*P* < 0.4295
Oesophageal cancer diagnoses	6.404	*P* < 0.041*	2.876	*P* < 0.237

IBD = inflammatory bowel disease

*Statistical significance

## Discussion

In this study, we evaluated the resilience of the Swedish health system by analysing changes in essential health services utilisation before, during and after the COVID-19 pandemic and by examining sociodemographic differences in utilisation by education and sex. All sectors that were assessed experienced initial disruptions. The analysis of service utilisation over time revealed a mixed picture: Primary care, as indicated by new prescriptions for type 2 diabetes, initially declined but recovered rapidly. Inpatient care for IBD experienced a reduction in admissions, although the duration of hospital stays remained stable. Emergency care, reflected by number of appendicitis diagnoses, showed a surprising increase in cases during the pandemic. Cancer diagnostics were significantly affected, with cervical cancer diagnoses declining markedly throughout the pandemic, while oesophageal cancer diagnoses rebounded swiftly after an initial drop. When examining sociodemographic differences, disruptions varied by education and sex but were generally not large.

The results of this study are, to some extent, supported by previous research. One study covering ten different countries assessed changes in outpatient visits, emergency room visits, inpatient admissions, surgeries and trauma admissions during the pandemic finding significant disruptions across a wide range of health services [24]. Two more in depth studies from Mexico showed that care for patients with diabetes and hypertension was severely impacted, which in turn led to a decrease in patients with these conditions under control [22].

A decrease in primary care utilisation during the pandemic has been seen in several other contexts [7, 22, 24]. In terms of service utilisation over time, new prescriptions for type 2 diabetes decreased sharply at the beginning of the pandemic, but with a rapid recovery to near pre-pandemic levels. This pattern indicates that Sweden’s primary care system held up against the pandemic’s challenges. The initial drop does not necessarily indicate a failure of the health system but can rather be interpreted as its ability to adapt and adjust in times of crises and postpone non-emergency care, which is itself a form of resilience. An additional factor to consider is the role of competing risks. Excess mortality during the pandemic, particularly among older adults, may have reduced the pool of individuals at risk of developing conditions such as type 2 diabetes and might potentially partly explain the decline in new prescriptions we observed.

An analysis of sociodemographic differences revealed a more nuanced picture. When stratified by education, a more pronounced drop in primary care utilisation was observed among individuals with lower education. This could be explained by barriers such as limited access to healthcare services, lower health literacy, or financial constraints, which may in turn reduce awareness of available services, decrease the frequency of healthcare, or complicate navigation the healthcare system. By contrast, differences between men and women were small, suggesting that sex did not play a major role in primary care utilisation during the pandemic.

Inpatient care results are consistent with prior studies in the US, UK, and Germany reporting significant disruptions in inpatient care [40–42]. An analysis of service utilisation over time in our study showed a decrease in the number of hospital admissions for IBD during the pandemic, while the average length of hospital stays remained stable [43]. Similar disruptions were observed in Germany, where children’s hospital admissions decreased by 20% in both 2020 and 2021, In comparison, our data show smaller declines in IBD admissions. The sustained decrease in IBD admissions even after the end of the pandemic could indicate that the system failed to recover from the initial shock, or it could simply be reflecting that the system was able to adapt. One explanation might be that health care staff learned to shift some inpatient care to outpatient care during the pandemic and that this shift was appreciated by staff and / or patients and therefore sustained. Another possibility is the increased use of telemedicine, which may have enabled some IBD patients to manage their conditions without hospital admission. Improved medical therapies introduced during the pandemic might have reduced the need for surgical interventions, thereby decreasing inpatient admissions. Stricter adherence to medications or lifestyle changes by patients to avoid flare-ups, possibly motivated by a fear of contracting a COVID-19 infection in the hospital, could also have played a role. Similarly, some patients, particularly those on immunosuppressive therapies, may have avoided hospitals altogether due to concerns about COVID-19 exposure. Additionally, limited access to diagnostic services during the pandemic might have delayed the identification of IBD flare-ups or complications, contributing to fewer hospitalisations. There was no change in the length of hospitalisation for IBD patients and thus no sign of more prevalent early discharges due to pandemic pressure on health care. A few studies have shown a possible link between IBD and either COVID-19 infection itself or COVID-19 vaccinations [44–46], but our results do not show any indication of such a link affecting inpatient care. Another possible explanation for the drop in hospitalisations is again the role of competing risks. Some studies suggest that patients with IBD have an increased risk of severe COVID-19 outcomes and higher mortality [47]. As a result, some patients may have died earlier in the pandemic, thereby reducing the number of individuals at risk of subsequent IBD-related hospitalisations. However, this effect is likely to be smaller than the changes attributable to care adaptations and patient behaviour.

When examining sociodemographic differences, distinct patterns emerged. When stratified by education, the higher education group had a lower rate of hospitalisation, which may reflect better disease management or lower incidence of the disease in this group in general. Individuals with higher education levels may be more proactive in managing their health, leading to earlier detection of complications. The drop in cases in line with the start of the pandemic was more pronounced in the higher education group indicating that this group may have been more worried about being exposed to the virus during a hospitalisation and therefore taking further precautions to avoid hospitalisation. Despite these differences in hospitalisation rates, the length of hospitalisation for IBD remained unchanged for both groups, indicating that the severity of cases, once admitted, did not differ significantly between the education groups. Although women generally had higher admission rates for IBD than men, the pattern of decline during the pandemic was similar for both groups. This suggests that the pandemic’s effect on inpatient care utilisation was not strongly sex-dependent, but rather influenced by other factors such as education and system adaptations.

The pattern of service utilisation over time in emergency care contrasts with reports from other countries. Although there were some early indications from both Sweden and Greece that COVID-19 infection may have increased the risk of appendicitis [48, 49], other countries reported a decrease in appendicitis during the pandemic [50, 51]. In Denmark for example a nationwide study noted a decrease in the total number of appendicitis cases but a shift towards more severe cases seeking treatment [51]. Tentatively, Sweden’s non-lockdown approach, combined with a potential increased risk due to COVID-19, might have contributed to the observed increase in appendicitis diagnoses at the start of the pandemic, followed by a significant decrease at its end. Furthermore, pandemic-related stress and lifestyle changes, such as altered diets or reduced physical activity, could have contributed to a slight increase in appendicitis incidence. In countries with stricter restrictions, the pandemic may have appeared more serious, with the strict measures making the threat of the virus a more immediate reality. This likely heightened public awareness of the potential to overwhelm the healthcare system, and people might have been more cautious about seeking care for non-COVID-19 related conditions, such as appendicitis. The temporary increase in cases may be related to the rollout of COVID-19 vaccinations, which could have encouraged people to resume normal care-seeking behaviour despite the ongoing pandemic. If there is a biological mechanism at play between COVID-19 and the risk of developing appendicitis, then this means that the Swedish health system was able to adapt to the increased influx in patients, therefore, indicating that emergency care in Sweden managed to uphold utilisation of health services during the pandemic. An analysis of sociodemographic differences was limited to sex, as education was not included for this indicator due to the high proportion of minor patients. For the available data, the higher incidence of appendicitis among men compared to women was consistent throughout the study period, which is in line with the current literature on the topic [52], indicating that the pandemic did not alter underlying sex-related differences in disease occurrence or care-seeking for acute conditions. However, it was beyond this study to evaluate if the quality of care was upheld.

The service utilisation over time for cervical cancer diagnostics was severely disrupted. The sharp decline in cervical cancer diagnoses due to disrupted screening services aligns with global findings of disrupted cancer care during the pandemic [22–24, 37, 53]. For instance, studies in Mexico reported a decrease in the number of diagnoses of cervical and breast cancer diagnoses [22, 23], a trend similarly observed in research from Romania and the Nordic countries [37, 53]. At the start of the pandemic, 13 regions in Sweden completely paused screening services and reduced them in 5 other regions [54]. However, some regions, such as Stockholm for example [55], adopted at-home screening methods in July 2020 when the National Board of Health and Welfare issued new guidelines [54]. Among the healthcare sectors analysed in this study, cervical cancer screening appears to have been most heavily affected. Planning for alternative screening methods during health crises is part of upholding the resilience of the health system [34]. Although there is a slight observable recovery in number of weekly cervical cancer diagnoses after the end of the pandemic it does not reach pre-pandemic levels. An analysis of sociodemographic differences was limited to education, as this indicator exclusively affects women. Cervical cancer diagnosis rates decreased similarly in both higher and lower education groups after the start of the pandemic, indicating that the disruptions to screening services impacted all sociodemographic groups equally.

In contrast, the service utilisation over time for oesophageal cancer diagnostics was largely stable. Diagnostics seem to not have been affected as much as cervical cancer by the pandemic. A possible explanation for this is the severe symptoms that patients with this disease usually present with, facilitating its detection even during the pandemic. An analysis of sociodemographic differences revealed subtle patterns. For oesophageal cancer, men showed a more rapid recovery in diagnoses after the initial decline compared to women, who experienced a temporary increase during mid-pandemic. These findings suggest sex-specific dynamics in diagnostic pathways. When stratified by education, a decrease in diagnoses was observed in both higher and lower education groups, with the higher education group having an overall lower incidence of cases.

Overall, disruptions to healthcare utilisation during the pandemic differed somewhat by education and sex. A more pronounced drop in type 2 diabetes diagnoses was seen in the lower education group, while IBD admissions fell more steeply in those with higher education. Sex differences, on the other hand, were smaller overall but remained visible: for example, IBD admissions were higher among women throughout, appendicitis more frequent in men, and oesophageal cancer showed slightly different recovery patterns by sex. These findings align with earlier studies suggesting social gradients in delayed care may vary depending on diagnosis and healthcare level [56, 57].

Strengths of the present study include the nationwide population-based coverage of the registers included. The data from the Swedish population-based healthcare registries is considered highly reliable and with high coverage [58, 59]. ITS analysis to evaluate changes in health service utilisation across different healthcare sectors during the pandemic was used, offering the advantage of clearly identifying temporal changes and trends influenced by the pandemic. Most of the indicators for the different parts of the health sector are based on previous literature [22, 23, 32, 33, 37, 53], making them useful for comparing with previous studies, and to get an overview of health system resilience in these sectors. While income, occupation, and education are distinct and not interchangeable indicators of SES [60], education is the most stable SES proxy in Sweden during the pandemic, especially when compared to the volatility of income and occupation during that time. Limitations of this study include incomplete data for the first A10B prescription indicator for new diagnoses of diabetes type 2 since guidelines changed, making it an unreliable indicator beyond October 2020. In addition, changes in the registry data may reflect alterations in data collection practices during the pandemic, rather than actual changes in patient care. Moreover, our classification of time periods is simplified. For example, excess mortality remained elevated during what we defined as the post-pandemic period, and the pandemic period itself encompassed both a pre-vaccine and post-vaccine phase. These differences may have influenced healthcare utilisation patterns and should be considered when interpreting the results. It should also be considered that competing risks may have influenced our findings for chronic diseases such as type 2 diabetes as it is more common amongst the elderly and IBD, where excess mortality reduced the pool of individuals at risk of new diagnoses. However, this mechanism is less relevant for acute conditions such as appendicitis, which presents suddenly and typically requires immediate medical attention regardless of competing risks, or for cancer incidence, where the key influence during the pandemic was delays in screening and diagnostics rather than removal of individuals from the at-risk population. Further limitations include the selection of indicators from different sectors, as there might be other factors at play affecting the changes seen. Aggregating education into just two categories may overlook nuances between the seven distinct education groups, but this approach was adopted for simplicity in visualisation, with the cut-off point based on previous research [61]. Lastly, while using national-level data in a decentralised health system like Sweden’s may not capture regional differences in pandemic responses and outcomes, the existence of overarching national policies [62] likely contributed to uniformity in pandemic response strategies across regions.

Perhaps the most important lesson learned from the study of these indicators is how to continue cancer screening and plan ahead before the next health crisis hits. Further research is also needed to better understand whether changes in IBD admissions indicate a failure to recover from the initial shock or simply the system adapting and becoming more efficient in the aftermath of the pandemic. Additional research on potential biological mechanisms between COVID-19 and appendicitis would be valuable. If this were indeed the case, it could be relevant especially in countries that saw a decrease in appendicitis cases meaning that they missed even more cases than previously anticipated.

Exploring socioeconomic factors, such as occupation, country of birth, and income, could provide valuable insights into varying health-seeking behaviours and further clarify disparities in healthcare access and utilisation. While this study focuses specifically on Sweden, its findings may also be relevant to other countries that choose to adopt a pandemic response approach similar to Sweden’s in future crises.

Overall, the findings emphasise the importance of maintaining routine care during pandemics by adapting quickly to the initial shock to avoid long lasting effects on patients. The health system managed to a large degree cater for the diagnoses observed, independent of educational level. We highlight areas for improvement in crisis preparedness, emphasising the importance to learn from the COVID-19 pandemic to improve the health system’s resilience to be adaptable and to ensure utilisation of health services during future public health emergencies, and the need for future research.

## Data Availability

The data that support the findings of this study are available from SWECOV but restrictions apply to the availability of these data, which were used under license for the current study, and so are not publicly available. Data are however available from the authors upon reasonable request and with permission of SWECOV.
